# Interrogating global narratives of trans queerness. Well-being and agency? Or more stories of trans trauma?

**DOI:** 10.3389/fsoc.2024.1343117

**Published:** 2024-04-05

**Authors:** Mark Vicars, James Milenkovic

**Affiliations:** Institute for Sustainable Industries and Liveable Cities, Victoria University, Melbourne, VIC, Australia

**Keywords:** (anti-)trans(gender), trauma, discourse, (psycho)social, well-being studies involving human subjects

## Abstract

In the international contemporary discourse transgender individuals arguably have an increased presence within public media highlighting the visible diversity that constitutes the LGBTQI2S+ community. However, in response to the challenging of cisgendered normative assumptions there has been an unprecedented swathe of anti-trans measures executed through the frenzied repealing of rights and freedoms within the key arenas of legal, medical, sporting, and educative domains. This paper explores the intersections of pathologizing rhetoric that emplotted anti-trans and transphobic discourses within and across public consciousness. The quotidian presence of these discourses provoked in us a wondering about how evolving conceptual debate is constructing a trans inclusive global imaginary. In this paper we situate trans safety not as a singular concept, but rather a differentially experienced phenomenon that is related to and embedded in questions of bio power and privilege. As such, when we refer to a trans safety imaginary, we are not solely addressing protection from physical violence but also safeguarding against psychological and emotional vulnerability.

## Introduction: (un)well-being and belonging

An intensification of anti-trans sentiment has occurred within recent years, with focal points occurring around gender-affirming care for children, trans women’s access to cis women’s sport and ‘single-sex’ spaces (bathrooms, changing rooms, and shelters). A slew of legislation negating the rights, freedoms, and bodily movements of trans individuals have been ratified with sense of displacement focused on specific incidents and events that epitomize feelings of not belonging, and at times of even not existing. At the time of writing this paper, 85 out of 586 bills impacting transgender people in America had passed; 376 bills were active (Trans Legislation Tracker, 2023). In Kentucky, *Senate Bill 150* prohibits transgender students from accessing school bathrooms and locker rooms that correspond to their gender identity; obliges schools to *out* students who divulge their gender identity or sexual orientation to their parents; does not require the recognition of the students’ pronouns ‘*that do not conform to a student’s biological sex*’; and prohibits health care providers from prescribing puberty blockers, cross-sex hormones, or perform ‘*sterilizing surgery*’ or gender-affirming surgery (Higdon, 2023). In Florida, *House Bill 1,069* prevents school employees and students ‘to refer to another person using that person’s preferred personal titles or pronouns if [they] do not correspond to that person’s sex’, stating that: ‘a person’s sex is an immutable biological trait and that it is false to ascribe to a person a pronoun that does not correspond to such person’s sex’ (“CS/CS/HB 1069: Education”, 2023). Discussion and instruction on gender and sexuality is now prohibited in classrooms from prekindergarten (pre-K) to grade 8. The Bill also states that schools cannot withhold information from parents ‘affecting a student’s mental, emotional, or physical health or well-being’ (“CS/CS/HB 1069: Education”, 2023), which may include gender identity and/or sexual orientation. Equally, Florida’s *Senate Bill 254* bestows powers to courts to remove children from their parents ‘if the child has been subjected to or is threatened with being subjected to sex-reassignment prescriptions or procedures’ (“CS/SB 254: Treatments for Sex Reassignment”, 2023). The Bill criminalizes access to gender affirming medications and surgeries to youths under 18 years, and makes their prescription a first degree misdemeanor for healthcare providers (“CS/SB 254: Treatments for Sex Reassignment”, 2023).

Challenges to trans people’s participation in social life are routinely underlined by medical/biological facticity. For example, in March 2023, the World Athletics Council announced that transgender women were precluded from female competitions if they had undergone male puberty (World Athletics, 2023). This is attributable to ‘certain aspects of male physiology’ (Bianchi, 2017, p. 229)—namely testosterone—being perceived as an unfair advantage leading to the outperformance of cis-women. Of course, biological discourse is already politicized (Zanghellini, 2020) and there is fervid debate on the definition of sex (Butler, 1990, 2004; Fausto-Sterling, 1993; Jackson and Scott, 2001; Eckert and McConnell-Ginet, 2013; Štrkalj and Pather, 2021; Ritz and Greaves, 2022).

Anti-gender/trans campaigners have attempted to homogenize the heterogeneity of gender/trans theory(ies) through caricature. For instance, the frequently deployed terms *gender ideology* and *trans ideology* denote monolithic onto-epistemologies, when there are many diversified conceptualizations. For example, Butler’s (1990) notion of gender performativity positions gender not as an internal reality but something that is accrued and coalesces into an identity through repetition. Yet, gender performativity runs in opposition to some trans theorists’ gender episteme whereby ‘the body or the materiality of gender has a kind of stubborn persistence’ (Halberstam, 2018, p. 59). Halberstam (2018) critiques that viewing gender within a performative frame infers an illegitimacy of trans genders, ungrounded in the ‘real, material and authentic (Halberstam, 2018, p. 120).

This paper pursues an examination of the material effects resultant from the persistent dissemination of trans and gender criticality within the public arena, with particular attention being paid to how transgender individuals face over four times the likelihood of experiencing violent victimization compared to cisgender individuals (Arantes and Vicars, 2023) exploring trauma not only as a psychological phenomenon, but also a sociological occurrence (Thompson and Walsh, 2010). Bettcher (2013) offers a ‘multiple meaning view’ (p. 234) of transgender onto-epistemology, questioning ‘singular fixed meanings of gender terms’ (p. 247), i.e., man and woman, utilized by the dominant culture. A core aim of this paper, is to generate critical insight into how materially produced injustices perpetuate cycles of disadvantage for individuals who are trans identified or identifying. We have sought to express Onwuachi-Willig’s (2016) argumentation that the circulation of increasingly nocuous anti-trans discourses has mediated a cultural trans trauma, and in doing so deconstruct the intersectionality of power and positionality to critically articulate the totemic power of cisgendered heteronormative expression and oppression(s) relevant to a wider readership seeking an introduction and understanding of the topic.

To craft frameworks for trans and gender diversity requires a venture into uncharted territory where weaving new threads of meaning and significance are yet to happen. Underpinned by a social justice praxis the analysis and discussion components develop a thematic coherence to reimagine the capacities for trans-identified individuals to actively pursue emancipatory dialogue. While *trans* as a signifier falls short in articulating their gendered sense of self, both authors are cognizant that those whose gender identities, variances, and expressions are disparate from their sex assigned at birth are umbrellaed under *trans* (Kuper et al., 2012; Williams, 2014; Halberstam, 2018). Thinking with deconstruction, Jackson and Mazzei (2012) tell us that ‘[a]ssuming a deconstructive stance is to both use and trouble categories at the same time’ (p. 20). Author 2 moves in and across the trans signifier vis-à-vis *strategic essentialism* (Spivak, 1996 [1985]), purposely locating themself under *trans* for the purposes of disrupting the discursive regime of cisnormativity. Author 1 identifies as a gay/queer man and in travelling across lines of difference situates re-centering to reframe and remake meaning and articulate how the ecologies of positionality, power, relationships and affect and experienced and re-negotiated and infused with productive relationality. The conclusion of this paper considers how the wider epistemologies of the gender critical movement have emplotted anti-trans ideology in the media and political domain to produce narratives of (trans) cultural trauma in public discourse.

## Articulations of (trans)gender and naming our researcher selves

Said (1975) has noted how culture exerts pressures and how it creates the environment and the community that allows people to feel they belong. Numerous cultures conceive of diverse expressions of gender and gendered presentation beyond the Western ontological assumption of the male/female binary. The Samoan *Fa’afafine* are assigned male at birth but do not inhabit the category of, nor are they recognized as, male (Vasey and Vander Laan, 2021). In India, *hijras* include those who are intersex or transgender, but also not male or female (Diehl et al., 2017). The *muxes* of Mexico are born male but adorn Zapotec garments and feminine accoutrements such as makeup and long hair (Diehl et al., 2017). The Indonesian *waria* are transgender women who may retain their male bodies whilst believing they ‘have the heart and soul of a woman’ (Toomistu, 2022, p. 73). The indigenous American *two-spirit* refers to persons born with both masculine and feminine spirits, and describes the intersection of gendered, sexual, and spiritual identities (Sheppard and Mayo, 2013).

It is therefore important to distinguish that terms like *gender* and *trans(gender)* are etymologically Western and operate within evolving historical, geographical, social, and cultural contexts. One must be cognizant of the monolingualism in inserting the foreign term(s) within non-English languages (Widerberg, 1998; Butler, 2021a). For instance, Widerberg (1998) reminds us that ‘where English is not the native language…there might be one…several or no words for gender’ (p. 134), and later: ‘it would seem that specific understandings of gender within most cultures cannot be properly translated; they get made into something else, into the understandings of gender that are implicit in the English language’ (p. 134). As terminologies have been formed within the colonial/modern world-system imagery and language there is an erasure of multiple subaltern subjectivities and different articulations of power and resistances. The paradox(es) of colonized identities, race and belonging inevitably confront sense and sensibilities of (un)belonging and place(lessness). Articulating counter narratives that contest a hegemonic narrative involves being reflective and reflexive of one’s researcher positionality owing to its emplottment within gendered, sexual, cultural, and racialized relations (Charmaz et al., 2018; Erickson, 2018). And ‘[a]lthough it is… not possible to name exhaustively all of the conscious and unconscious baggage that the researcher brings [to the research] [here, we offer a] comprehensive statement of [our] … epistemological orientation [and] social positionality’ (Scheurich, 1995, p. 249).

## The anti-gender movement

It is useful to locate anti-trans rhetoric within the wider epistemologies of the gender critical movement. Opposing what is labelled *gender ideology*, this reactionary group includes the Right, religious conservatives, including the Catholic, Evangelical, and Pentecostal churches, and nationalist groups (Loughlin, 2018; Butler, 2021b; Hsu, 2022; Venegas, 2022). The Vatican, for example, is a staunch opponent of gender theory, enshrining the complementarianism of male and female and dimorphic sexuality as essential for the preservation of the traditional family (Loughlin, 2018; Butler, 2021b; Sosa, 2021; Venegas, 2022). In some countries, *gender ideology* is viewed as a cultural import that threatens values and ways of life (Paternotte and Kuhar, 2018; Sosa, 2021; Butler, 2021a): imperiling the family, masculinity, and femininity (Venegas, 2022). Anti-gender campaigners have regarded *gender ideology* as an insidious plot to impose ‘deviant and minority values’ (Paternotte and Kuhar, 2018, p. 9) on all, but particularly children.

The anti-gender campaign, which Butler (2021b) labels ‘nationalist, transphobic, misogynist, and homophobic’, has become a transnational mobilization, with exponents in Germany, Poland, Brazil, Italy, east Asia, Columbia, and Costa Rica (Butler, 2021b). Some have observed the goal of anti-gender movements as stripping basic human rights and democratic projects (Sosa, 2021; Butler, 2021b), for example, ‘intimate/sexual citizenship, including LGBT rights, reproductive rights, and sex and gender education’ (Paternotte and Kuhar, 2018, p. 8). This also includes feminism, same-sex marriage, same-sex adoption, abortion, non-essentialist discourses on gender and sexuality, and trans rights (Butler, 2021b; Venegas, 2022). Scholarship has traced the proliferation of trans-related matters within the contemporary zeitgeist. From trans women’s access to single-sex spaces (Herman, 2013; Schilt and Westbrook, 2015; Barnett et al., 2018; Davis, 2018), trans women’s participation in cis women’s sports (Sykes, 2006; Schultz, 2011; Love, 2014; Bianchi, 2017; Sailors, 2020), *sex-change regret*/de-transitioning (Temple Newhook et al., 2018; Hildebrand-Chupp, 2020; Slothouber, 2020; MacKinnon et al., 2021; Turban et al., 2021), *trans/gender ideology* allegedly indoctrinating children (Westbrook and Schilt, 2014; Pearce et al., 2020), the related trans *social contagion* and *conversion* of cisgender gay/lesbian youth to heterosexual trans youth (Marchiano, 2017; DeLay et al., 2018; Littman, 2018; Shrier, 2020; Soh, 2020; Ashley, 2020a,b), and the medicalization of children via puberty blockers and cross-sex hormones (Cohen-Kettenis et al., 2011; Ashley, 2019; Priest, 2019; Giordano and Holm, 2020; Zanghellini, 2020; Rew et al., 2021). This corpus of research speaks collectively to what has been labelled *the culture war*, *the trans issue*, and *the woke agenda*.

## Emplotted anti-trans ideology

Goffman (1963) has highlighted how stigma is an enduring condition that can actively discredit individuals and he coined the phrase ‘stigmaphile’ to name the space of the stigmatized and ‘stigmaphobe’ to refer to the world of ‘normalcy’. Warner (2000) has noted how:

The stigmaphile space is where we find a commonality with those who suffer from stigma, and in this alternative realm learn to value the very things the rest of the world despises…The stigmaphobe world is the dominant culture, where conformity is endured through fear of stigma (p. 43).

The quotidian presence of these discourses provoked for us a wondering about bodies that are defined ‘by their potential to reciprocate or co-participate in the passages of affect’ (Seigworth and Gregg, 2010, p. 2), and how such ‘bodies are shaped by their dwellings and take shape by dwelling’ (Ahmed, 2006, p. 9). The nexuses between the epistemology of what Savage (2007) calls *the outgroup*—the Other—in the nineteenth century and contemporary anti trans discourse we have italicized within the below quotation:

[the] “scientific” reason as to why outgroups were inferior and other-than-human (legitimizing their destruction)[*refusing the biological body, ‘reality’ denialism*], as well as threatening (necessitating it)[*a danger to women, children, society at large*], but also a metaphorical representation, revolving around concepts of hygiene and purity [*hormones and surgical evisceration the body*], which fulfilled identical psychological necessities on a symbolic and populist level…the emergence of the nation-state [*sweeping anti-trans legislature*] allowed perpetrator groups to conceive of themselves as a unitary body [*the anti-trans/gender movement*] within defined geographical limits whose ideal state was one of “racial”[*cisgendered*] homogeneity. The indelible “wrongness” of an outgroup necessitated, in the eyes of perpetrators, the complete removal of that group from a geographically bounded territory [*women’s bathrooms/sports, schools*]; a particularly vicious medicalized representation of outgroups as a biological threat not only *legitimized* their disappearance, but directly *motivated* it’ (Savage, 2007 pp. 404–405).

Vis-à-vis the ever-tightening political, social, educative, and medical disciplinarity of cisgenderism Foucault’s (1988) *biopolitics* is a useful lens through which to consider how the State ‘wields its power over living beings’ (p. 160) and how the diaspora of anti-trans sentiment becomes material as a dialogical relation, and its affects becomes *sticky* (Ahmed, 2010). By this we mean any ontological assault on the ‘metaphysical “transgender” identity itself’ (Brown, 2021, p. 188) are socially rewarded and transgressive behaviors are punished’ (Green (1998, p. 26). We argue that the rescinding of rights and the implementation of restrictions removes trans people from the province of intelligibility, rendering them non-human and engenders a socially embedded trauma. Hamburger (2017) defines social traumas as those where ‘the whole of the social environment is under threat of persecution or actually experiencing persecution’ (p. 80). This is evinced through legislation directly affecting trans persons. Mizock and Lewis (2008) convey that transphobia and violence produce experiences of trauma and associated mental health issues. Risk factors correlated with trans trauma include post-traumatic stress disorder (PTSD) and acute stress disorder (Mizock and Lewis, 2008). To palliate this trauma, risky behaviors such as alcohol and substance abuse, and engaging in risky sexual practices might ensue (Hall and DeLaney, 2021). Equally, ‘[s]uicidal ideation in response to transphobic trauma is a critical concern for transgender individuals’ (Mizock and Lewis, 2008, p. 342). Richmond et al. (2012) add that ‘having a doctor ask inappropriate or invasive questions during a routine check-up or hearing a talk show host discuss trans issues in pathological or demeaning ways’ (p. 47) are trauma-triggering. Hall and DeLaney (2021) add that a ‘[transgender] individual who has problems using gender-segregated restrooms or who suffers relentless misgendering everywhere they go might avoid leaving the house altogether to minimize physical discomfort and instances of identity abuse’ (p.1279). Indeed, Butler (1990) identifies that when ‘[c]ertain humans are recognized as less than human…that form of qualified recognition does not lead to a viable life’ (p. 2).

## Psycho-social trauma

Thompson and Walsh (2010) remind us of the significance of acknowledging the ‘psychological and sociological dimensions’ of trauma (p. 379). Theorizing with Heidegger’s (1962) *being-towards-others* as a framework to diverge from the localized and individual experience of trauma Thompson and Walsh (2010) to perceive trauma as a psycho-social phenomenon that embraces an: ‘intercorporeality and trans-subjectivity’ of bodies (Blackman and Venn, 2010, p. 8). Thinking with this scholarship not only speaks to the affective force that connects people (Helmsing, 2022) in ‘how the collective takes shape through the impressions made by bodily others’ (Ahmed, 2004, p. 27), but also directs attention to the wider politico-sociocultural relations (Thompson and Walsh, 2010). Onwuachi-Willig (2016) tells us that ‘cultural trauma arises because of a public or official sanctioning of the everyday denigration and subjugation of the subordinated group which reinforces a historicity that their rights are not protected and respected in society’ (pp. 336–337). Within this matrix, Erikson (1991) positions trauma as a mutual relation between a community through ‘situations where trauma becomes so widely shared within an existing collection of people that it dominates its imagery [and] governs the way members relate to one another’ (p. 461). It is apparent that trauma is not exogenous in its presentment. These affective states are triggered by ‘an assault from outside that break into the space one occupies as a person and damages the interior’ (Erikson, 1991, p. 455). These insidious traumas are ‘ongoing negative experiences associated with living as a member of an oppressed group’ (Szymanski and Balsam, 2011, p. 4). Likes waves protractedly wearing away at the cliff face, these traumas are ‘repetitive and enduring’ (Richmond et al., 2012, p. 47). Subsuming the atomized traumatogenic experiences of the trans body politic reveals a prevalent phenomenon: a (trans)cultural trauma.

## A (trans)cultural trauma lens

Onwuachi-Willig (2016) employed a *cultural trauma narrative* framework to the responses of the black community to the acquittal of two white men for the murder of a 14 year-old African-American youth. Onwuachi-Willig (2016) reasons that there are three criteria which indicate the emergence of cultural trauma:

First, there must be a longstanding history of the routine harm, a history that essentially leads the subordinated group to expect nothing other than the routine yet cultural trauma—inducing injury. Second…the routine injury must have garnered the type of widespread media attention that makes a large audience, both within and outside the subordinated group, take notice of the routine occurrence…. Third…there must be public discourse about the meaning of the routine harm, a harm that usually occurs in the form of governmental or legal affirmation of the subordinated group’s marginal status. (p. 336)

We repurpose this analytic to examine habitual encounters of harassment, violence, and stigmatization, together with deleterious reportage of trans people and issues and the revoking of rights, and how these constitute a cultural trauma. We expatiate upon each of Onwuachi-Willig’s (2016) criterions below.

### Criterion one: a longstanding history of the routine harm

Trans individuals, as liminal bodies outside of dominant cisgendered norms, have historically experienced sexualization, stigmatization, and psychopathologizing. The controversial phenomenon of autogynephilia—*love of oneself as a woman*—is one example. Psychologist Ray Blanchard coined autogynephilia in the late 1980s to describe how male-to-female transexuals (MtF) are either androphilic (homosexual transsexuals) or non-androphilic (aroused by the thought of becoming women) (Blanchard, 1989, 2005; Bailey and Triea, 2007). According to the autogynephilic thesis, *auto gynephiles* suffer a paraphilia, amongst which include criminal sexual behaviors like pedophilia (Serano, 2020). This diagnosis has offered opponents of trans rights recourse to pathologization in order invalidate trans women’s claim to womanhood (see Joyce, 2022). For example, Serano (2020) observes that autogynephilia has been weaponized within trans-exclusionary radical feminism (TERF) to ‘insinuate that trans women are merely ‘sexually deviant men” (763–764). It may be argued that pathologization, undergirded by mechanisms of power, contributes to *subjection*—the formation of a subject (Butler, 1997).

In order to gain access to gender-affirming care including puberty blockers, cross-sex hormones, and gender-affirming surgeries, trans individuals must, oxymoronically, accede to the pathologizing diagnosis of gender identity disorder (GID)—now gender dysphoria (Butler, 2004; Mizock and Lewis, 2008; Serano, 2016). Submitting to this psychological condition, as Butler (2004) states, ‘imposes a model of coherent gendered life that demeans the complex ways in which gendered lives are crafted and lived (p. 5). Contagion rhetoric, reminiscent of that used in anti-gay, lesbian, and bisexual discourse, has been rearticulated towards trans people. Contagion’s metaphoric power resides in its likeness to ‘the process of cultural transmission’ (Davis, 2002, p. 830), where ideas and attitudes are disseminated amongst the culture. Equally, contagion, goaded by fear, bestows onus to the Other for incipient disease (Pernick, 2002). As put by Hsu (2022), ‘[t]he language of contagion stokes panic associated with viral epidemics, demanding an urgent governmental and social response’ (p. 65). This scaremongering is conveyed through the phenomenon—the ‘psychic epidemic’ (Ashley, 2020a, p. 783)—of rapid-onset gender dysphoria (ROGD). This diagnosis posits that the deep dissatisfaction one feels about their gender emerges from a social contagion (Slothouber, 2020), with parents reporting online that their children, namely teenage girls, are suddenly, without their presentiment, becoming gender dysphoric (Littman, 2018). Littman’s (2018) work has garnered much criticism in terms of methodological rigor and interpretation (Restar, 2020; Bauer et al., 2022). Despite no clinical validation of this apparent subclass of gender dysphoria (World Professional Association for Transgender Health [WPATH], 2018), this diagnosis is given credence by the anti-trans lobby to validate a pervasive contagion amongst adolescent girls (see Shrier, 2020; Joyce, 2022).

In educational domains, LGBTQIA2S+ students experience disproportionately higher instances of bullying, marginalization, depression, anxiety, suicidality, academic difficulties, and truancy compared to heterosexual, cisgendered pupils (Walton, 2005; Almeida et al., 2009; Garron and Logan, 2020; Shevlin and Gill, 2020; Schreuder, 2021). Trans students often feel unsafe within the ambivalent ambience of cisgenderism inscribed in schools (Ryan and Hermann-Wilmarth, 2013). Shevlin and Gill’s (2020) discourse analysis on parental attitudes towards the Safe Schools program—an inclusive and anti-bullying program in Australia—discerned *transhysteria* as particularly conspicuous. One participant demonstrated apprehension in their child being perceived as trans through the wearing of pants. Panic and hysteria materialized around the use of toilets, with anxieties around assault engineering the narrative of the innocent child in need of protection and the sexualizing transgender bogeyman (Shevlin and Gill, 2020). Moreover, children and adolescents’ transgender identities were met with incredulity, with parents ‘positioned as omniscient dictators of identity’ (Shevlin and Gill, 2020, p. 906).

Scholarship reveals that trans individuals experience disproportionate incidences of sexual, physical and verbal violence (Koyama, 2003; Butler, 2004; Mizock and Lewis, 2008; Bettcher, 2013; Perry and Dyck, 2014; Halberstam, 2018; Pearce et al., 2020; Stanley, 2021; Suh, 2022); have trouble accessing, and abuse within, the healthcare system (Kidd and Witten, 2007), securing employment and housing (Mizock and Lewis, 2008), and homelessness (Wilkinson, 2014). Priest (2019, p.46) has noted, ‘[t]ransgender youth are 10 times as likely to attempt suicide when compared to their cisgender peers’ and that trans individuals have suffered, and continue to suffer, from a ‘constellation of systemic oppressions’ (Kidd and Witten, 2007, p. 52).

### Criterion two: widespread media attention

Concurrently, essentialist and pathologizing rhetoric from politicians, commentators, authors, and journalists resound and reverberate within and across public consciousness, leading to dis-information and the stoking of fears around what has been termed the *trans debate*. In March 2023, outspoken British gender/trans critic, Kellie-Jay Keen, toured Australia with her *Let Women Speak* tour. The Melbourne leg of the tour drew international attention when neo-Nazis, brandishing the banner DESTROY PAEDO FREAKS attended the event, performing the *Sieg Heil* salute on the steps of Parliament House (Kolovos, 2023).

Waites (2018) asks the question: ‘[w]hen, should we speak of identity erasure in global queer politics?’ (p. 44) as it is not only far right leaning, but voices also conveying antipathy. Splinter factions within the queer community have also voiced opposition to trans rights. In June 2023, a billboard reading ‘Let kids be kids’ was erected in Tasmania by trans and gender critical group *LGB Tasmania* (Woodall, 2023). This phrase refers to the purported indoctrination and sexualization of children vis-à-vis *trans ideology*. *LGB Alliance Australia* confers that ‘biological sex is observed in the womb and/or at birth and is not assigned’ and ‘current gender ideologies are pseudo-scientific and present a threat to people whose sexual orientation is towards the same sex…we believe that these ideologies are confusing and dangerous to children’ (LGB Alliance Australia, n.d.). Additionally, *Binary Australia* ‘campaigns for action against the radical gender ideology harming young people, women, and society’ (Binary Australia, n.d.), and ‘exists to challenge the aggressive agenda to remove sex from our society in the areas of education, health, military, business, politics and the law’ (Binary Australia, n.d.).

The anachronistic question *what is a woman?* has become a motto in anti-trans discourse propounded by some politicians in anti-trans discourse, having the metaphysical intent to question the materiality of the female body as ‘immobile, stable, coherent, fixed, pre-discursive, natural, and ahistorical’ (Moi, 1999, p. 4).

### Criterion three: public discourse about the harm

The politico-socio-cultural landscapes within Australia are becoming increasingly fraught with hostile anti-trans rhetoric. In 2020, One Nation MP, Mark Latham, introduced the *Educational Legislation Amendment (Parental Rights) Bill* which sought to contest and de-realize trans students while ‘prohibit[ing] the teaching of the ideology of gender fluidity to children in schools’ (Education Legislation Amendment (Parental Rights) Bill 2020 (Cth), n.d., p. 1). In 2022, former Liberal party candidate, Katherine Deves, worked with Tasmanian senator, Claire Chandler, in developing what became known as the *Save Women’s Sport Bill* (*“Sex Discrimination and other Legislation Amendment (Save Women’s Sport) Bill 2022* Cth”, n.d.), which sought to amend the Sex Discrimination Act to segregate women’s sports on the basis of biological sex.

In the U.S., department store *Target* removed particular Pride month merchandise from shelves following hostile responses from shoppers’ and physical confrontations with staff (D’Innocenzio, 2023). Such items include bathers/swimmers that allow pre-operative or trans women who do not desire the removal of their penis a provision to tuck their genitals. Transgender influencer and TikTok personality, Dylan Mulvaney, attracted condemnation and threats of boycott for her partnership with *Bud Light* (Holpuch, 2023), makeup brand *Maybelline* (Picchi, 2023), and for advertising women’s *Nike* apparel (Zilber, 2023). Former U.S. president Donald Trump’s 2024 presidential election pitch featured a platitude of proposed restrictive measures against transgender individuals—notably gender affirming care for trans children, including access to puberty blockers and gender affirmation surgeries. Additionally, Trump averred that he will promote schools championing the heteronormative family unit and introduce a Bill that recognizes male and female as the only genders recognized in the U.S. (Seitz-Wald and Yurcaba, 2023). Four years earlier in 2019, the Trump government officially forbad trans people from enlisting in military service (Jackson and Kube, 2019).

In December 2022, Scotland passed their Gender Recognition Reform Bill, which sought to streamline the process for trans people to legally change their gender. The Bill removed the need for the pathologizing diagnosis of gender dysphoria, reduced the age of eligibility for a gender recognition certificate from 18-to 16–17-year-old, and reduced the timeframe where one must live fulltime in their gender from two years to three months (Brooks, 2022). In January 2023, however, the U.K. government vetoed the Bill on the grounds of its discrepancies with the Equality Act 2010, notably around the site of sex as a protected characteristic (Equality Hub, Government Equalities Office, and Office of the Secretary of State for Scotland, 2023). In June 2023, England’s National Health Service (NHS) rescinded access to puberty blockers for youths questioning their gender, stating: ‘NHS England would in future only commission GnRH analogues [puberty suppressing hormones] in the context of a formal research protocol’ (NHS England, 2023a), and: ‘outside of a research setting, puberty suppressing hormones should not be routinely commissioned for children and adolescents who have gender incongruence/dysphoria (NHS England, 2023b). A myriad of literature supports the routinization and efficacy of puberty suppressing drugs (Cohen-Kettenis et al., 2011; Ashley, 2019; Priest, 2019; Giordano and Holm, 2020; Riggs et al., 2020; Rew et al., 2021).

## Conclusion—there are few words left with which to speak

The material-discursive effects of numerous anti-trans ideological enterprises resemble what Thompson and Walsh (2010) designate ‘an *existential* injury’ (p. 378): trauma experienced because of ‘an assault on the self’ (p. 378) which calls into question a person’s place within the fabric of social relations. *Existential injuries* destabilize the moorings of one’s existence, ‘inflict[ing] a wound to meaning’ (Nguyen, 2011, p. 28) and ‘severely altering how we see the world and how we make sense of it—in effect, destabilizing or even shattering our frameworks of meaning, our spiritual and existential foundations’ (Thompson and Walsh, 2010, p. 379). Whilst trans inclusion and inclusive practices have become a popular international buzzword, a scar is being left on the psyches of trans and gender diverse people who have been and are being traumatized by and in their realities and lived experiences. Research has examined the association between anti-trans sentiment and *exterminationist politics* (Owen, 2022) and Panter (2020) adduces that ideologies of erasure share the objective of attempting to exterminate ‘a specific targeted group who violate social, theological, and politically enforced heteronormative expectations’ (p. 81). Brown (2021) has described the systematic murders of trans people—particularly trans women—as ‘contain[ing] a genocidal logic…demonstrative of the eliminationist intent inherent to both life force atrocities and genocide’ (p. 186). This gains salience when considering 2021 had the highest murder rate of transgender people worldwide with 375 deaths (Powell, 2021; Trans respect versus Transphobia Worldwide, 2021), and in 2018, 167 transgender people were killed in Brazil alone (Brown, 2021).

Within this study, the intersections of power that converge on transgender lives shaped by the associated religious, political ideologies and their aligned authoritative bodies (Fyfield, 2022) in turn have impacted legal and legislative frameworks that provide a foundational ground of anti-trans operations (Cumming-Potvin and Martino, 2018). Media discourses which operate within their own intersections of power, influence emergent gender dysphoria and the resultant mental health impacts of inaction (Zaliznyak et al., 2021). In final summary, there is a growing need for allies to advocate for transgender and gender diverse individuals, as reactionary right-wing political forces in the anglosphere are trying and/or succeeding to pass and enforce discriminatory and disciplinary legislation (Vicars and Wolfe, 2023).

## Author contributions

MV: Writing – original draft. JM: Writing – original draft.
